# Increased Adiposity Enhances the Accumulation of MDSCs in the Tumor Microenvironment and Adipose Tissue of Pancreatic Tumor-Bearing Mice and in Immune Organs of Tumor-Free Hosts

**DOI:** 10.3390/nu11123012

**Published:** 2019-12-10

**Authors:** William J. Turbitt, Shawntawnee D. Collins, Huicui Meng, Connie J. Rogers

**Affiliations:** 1 Department of Nutritional Sciences, The Pennsylvania State University, University Park, PA 16802, USA; billturbitt@gmail.com (W.J.T.); collinss217@gmail.com (S.D.C.); menghc@mail.sysu.edu.cn (H.M.); 2 Penn State Cancer Institute, The Pennsylvania State University, Hershey, PA 16802, USA

**Keywords:** diet-induced obesity, calorie restriction, immunosuppression, survival, adipose tissue

## Abstract

Obesity is associated with increased risk and reduced survival for many types of cancer. Increasing adiposity may affect the balance between immunosuppressive and antitumor mechanisms critical for dictating cancer progression or remission. The goal of the current study was to determine if increased adiposity altered tumor growth, survival, and myeloid-derived suppressor cell (MDSC) accumulation in a subcutaneous murine model of pancreatic cancer. C57BL/6 mice were placed on a 30% kcal calorie-restricted diet, 10% kcal from fat diet fed ad libitum, or 60% kcal from fat diet fed ad libitum for 16 weeks to generate lean, overweight, and obese mice, respectively; followed by subcutaneous injection with 1 × 10^6^ Panc.02 cells. We observed a significant linear relationship between increased adiposity and increased tumor growth and mortality; increased accumulation of Gr-1^+^CD11b^+^ MDSCs; and reduced CD8 T cell:MDSC ratio in multiple tissues, including tumor. Increased adiposity also increased the accumulation of MDSCs in the spleen and lymph node of tumor-free mice. These data suggest adiposity induces MDSC accumulation, which may contribute to an immunosuppressive environment promoting tumor growth. Overall, our findings provide a rationale to prevent or reverse increased body weight as a strategy to reduce the accumulation of immunosuppressive cell types.

## 1. Introduction 

Cancer is a leading cause of death worldwide [1]. Obesity increases the risk of cancer at 13 sites, including breast, colon, and pancreas, among others [2,3]. Obesity is also associated with accelerated tumor progression, poorer response to treatment, and increased cancer-related mortality in multiple cancer types including pancreatic and breast cancer [4,5,6]. In addition to the well-established relationship between obesity in adulthood and poorer cancer outcomes, being overweight or obese during early life may also increase subsequent mortality risk for certain cancer types, including pancreatic cancer [7]. Emerging evidence suggests that survivors of childhood cancer experience significant weight gain early during the treatment window that is maintained after treatment concludes [8,9,10]. Survivors of childhood cancer also have a six-fold higher risk of developing second malignant neoplasms [11,12], and obesity during childhood cancer treatment contributes to this risk [13]. Thus, gaining a better understanding of the mechanisms linking obesity and cancer may lead to novel strategies to intervene and improve clinical outcomes in numerous cancer types.

Several possible mechanisms, including immune modulation, may underlie the relationship between having obesity early in life and/or throughout adulthood on cancer risk, progression, and mortality. Obesity can promote tumor growth by modulating the balance between protective antitumor mechanisms and tumor-promoting/immunosuppressive factors [14,15,16]. Obesity impairs the function of cytotoxic CD8^+^ T cells and natural killer (NK) cells, two cell types with potent antitumor effector functions [17,18]. Obesity can also increase the immunosuppressive environment associated with tumor progression further blunting antitumor immune mechanisms. In particular, obesity-induced changes in inflammatory mediators may contribute to the expansion, accumulation, and activation of myeloid-derived suppressor cells (MDSCs) [19]. MDSCs are a diverse population of immature myeloid-lineage cells that accumulate in cancer, autoimmunity, and some chronic inflammatory conditions [20,21,22]. MDSCs can suppress cells of both the innate and adaptive immune system, including NK cells, macrophages, dendritic cells (DCs), and, in particular, T cells, via multiple mechanisms [23]. The accumulation of MDSCs correlates significantly with cancer stage and severity, and often coincides with a concurrent reduction in CD8^+^ T cells [24,25,26]. The mechanisms by which the expansion of adipose tissue contributes to the accumulation and function of MDSCs in tumor-free and tumor-bearing hosts remains unknown.

Pancreatic adenocarcinoma (PDAC) is an aggressive tumor type, with an overall five-year survival rate of 7% [27]. The infiltration of effector CD8^+^ T cells into the tumor confers a survival benefit and is predictive of a beneficial response to therapy; however, CD8^+^ T cells are found infrequently in the tumor microenvironment (TME) of pancreatic cancer patients [28,29,30]. In contrast, myeloid cells are highly prevalent in the TME, play a significant role in immune suppression, and are correlated with poor responses to therapy [31,32]. Obesity promotes an increase in circulating inflammatory cytokines and alters the infiltration of immune cells in PDAC, which is associated with increased tumor growth and metastasis [33,34]. However, additional studies are needed to determine if increasing adiposity changes the accumulation and function of immune suppressive myeloid cells.

Evaluating the role of obesity on the emergence of MDSCs in a murine model of pancreatic cancer may provide critical insight into the link between obesity, immune suppression, and poor clinical outcomes. Therefore, the goal of the current study was to determine if increased adiposity altered tumor growth, survival, and MDSC accumulation and function in a subcutaneous murine model of pancreatic cancer. This model was chosen because obesity exacerbates tumor growth [33,35], and we [36], and others [37], have demonstrated a role for the immune system in controlling Panc.02 tumor growth. An additional goal of this study was to determine if obesity alone (in the absence of tumor) enhanced the accumulation of MDSCs in relevant lymphoid organs.

## 2. Materials and Methods

### 2.1. Tumor Cell Line and Cell Culture

The murine ductal adenocarcinoma cell line Panc.02 was kindly provided by Dr. Jeffrey Schlom (National Cancer Institute, National Institute of Health). Panc.02 cells were cultured in McCoy’s 5A without G418 (ThermoFisher Scientific; Waltham, MA, USA), and supplemented with 10% FBS (Gemini BioProducts; Sacramento, CA, USA), 0.1 mM nonessential amino acids (Mediatech; Manassas, VA, USA), 1 mM sodium pyruvate (Mediatech), 2 mM glutamine (Mediatech), 10 mM HEPES (Mediatech), 100 U/mL penicillin streptomycin (Mediatech). Panc.02 tumor cells were maintained at 37 °C and 5% CO_2_ and passaged every 3–4 days using trypsin/EDTA (Mediatech). 

### 2.2. Animal Model 

Female 6-week-old C57BL/6 and BALB/c mice were obtained from Jackson Laboratory (Bar Harbor, MA, USA). All mice were housed at the Pennsylvania State University and maintained on a 12-h light/dark cycle with access to food and water. One cohort of mice (*n =* 90) was fed a semipurified control diet (D12450B, Research Diets, Inc., New Brunswick, NJ, USA) and were used to characterize the growth rate of Panc.02 tumors, evaluate the time course of Gr1^+^CD11b^+^ MDSC accumulation, and assess the function of MDSCs in this model. A second cohort of mice (*n =* 130) were randomized to receive one of the following diets (all purchased from Research Diets, Inc.) for 16 weeks: (i) a control diet containing 10% kcal from fat (D12450B; consumed ad libitum); (ii) a calorie-restricted (CR) diet (D03020702), a modified AIN-76A semipurified diet fed in daily aliquots to provide 30% less total energy and 100% of all vitamins, minerals, fatty acids, and amino acids relative to the control group; or (iii) diet-induced obesity (DIO) diet (D12492; consumed ad libitum), a modified (60 kcal% fat) AIN-76A semipurified diet providing approximately 30% more total energy with 100% of vitamins, minerals, and amino acids, relative to the control diet. Diet formulations are shown in Supplementary Table S1. A subset of mice on each diet (*n =* 12–14 per group) were removed from the study prior to tumor injection to evaluate body composition, metabolic markers and immune cell distribution. All remaining mice continued on their respective diets following tumor implantation. Food intake and body weight were monitored as previously reported [38], and mice were observed daily for signs of ill health. Animal care was provided in accordance with the procedures outlined in the "Guide for the Care and Use of Laboratory Animals." The Institutional Animal Care and Use Committee of the Pennsylvania State University approved all animal experiments (IACUC protocol number 42335). 

### 2.3. Tumor Protocol

Panc.02 cells (1 × 10^6^) were suspended in PBS and injected s.c. into the lumbar region of mice. Tumor growth was monitored three times per week with a digital caliper from day 13 post-tumor implantation until 60 days post-tumor implantation or when mice met criteria for removal of study (i.e., tumor volume exceeded 1.5 cm^3^ or animals were moribund). Tumor volume was calculated by multiplying the short side × short side × long side/2 × 0.001 to get tumor volume in cm^3^. 

### 2.4. Immune Cell Depletion

C57BL/6 mice (*n =* 10–11/group) were implanted s.c. with 1 × 10^6^ Panc.02 cells. Mice were injected i.p. with saline, 100 mg/injection isotype control (clone LTF-2; BioXCell; West Lebanon, NH, USA), or 100 mg/injection anti-Gr-1 (clone RB6-8C5; BioXCell) antibody every three days beginning at day 16 post-tumor implantation. Mice were sacrificed at day 40 post-tumor implantation. 

### 2.5. Body Composition Analysis

Mouse carcasses were scanned using a GE Lunar PIXImus Dual-Energy X-ray Absorptiometer (DEXA) to assess lean mass, fat mass, and percent body fat, as previously described [39].

### 2.6. Isolation of Spleen, Lymph Node and Tumor-Infiltrating Immune Cells

Spleens, tumor-draining lymph nodes (TDLN), and tumors were harvested, and single-cell suspensions were prepared as previously described [6,40]. Cell counts and viability were determined via trypan blue exclusion (Corning; Tewksbury, MA, USA).

### 2.7. Flow Cytometric Analyses

Single cell suspensions of splenocytes, TDLN, and tumor-infiltrating immune cells were washed twice in PBS containing 0.01% bovine serum albumin at 4 °C. Cells were incubated with Fc block (Biolegend; San Diego, CA, USA) and stained with saturating concentrations of conjugated antibodies, listedin Supplemental Table S2, as previously described [6,40]. Lymphoid and myeloid cells were gated on forward vs. side scatter, and a total of 30,000 events were acquired. Flow cytometric analyses were performed on a Beckman Coulter FC500 flow cytometer (Beckman Coulter; Indianapolis, IN, USA). Flow cytometric analyses were plotted and analyzed using Flow Jo software (Tree Star; Ashland, OR, USA). 

### 2.8. MDSC Isolation

MDSCs were isolated from a single cell suspension of splenocytes as per the manufacturer’s instructions (MSDC Isolation kit; Miltenyi Biotec; Gladbach, Germany). Post-isolation MDSC subsets (Gr-1^Hi^Ly6G^+^ cells and Gr-1^Lo^Ly6G^-^cells) were counted and viability was determined via trypan blue exclusion. 

### 2.9. T Cell and APC Isolation

T cells were isolated via negative selection using the Dynabeads® Untouched^TM^Mouse T cells Kit (Life Technologies; Carlsbad, CA, USA). APCs were isolated using the Dynabeads® Mouse Pan T (Thy1.2) Kit (Life Technologies). After isolation, T cells and APCs were counted, and viability was determined via trypan blue exclusion. 

### 2.10. Mixed Lymphocyte Reaction 

Isolated splenic T cells (1 × 10^5^) from BALB/c animals were cocultured with irradiated (2000 rads) APCs (5 × 10^5^) isolated from Panc.02 tumor-bearing C57BL/6 mice on each of the diets (*n =* 7–8/group), and either granulocytic (Gr-1^high^Ly6G^+^) or monocytic (Gr-1^dim^Ly6G^-^) MDSCs (5 × 10^4^). Cells were incubated in flat-bottomed, 96-well plates and were pulsed with 1 μCi per well of tritiated thymidine at 96 h. Proliferation was assessed by tritiated thymidine (H^3^) incorporation at 120 h (Perkin Elmer; Waltham, MA, USA) on a microbeta plate reader (Perkin Elmer). Each assay was performed in triplicate.

### 2.11. Gene Expression

Gene expression of *Gr1*, *Il6*, *Il10*, *Cd3*, *Arg1*, and *Tgfb* was assessed in the adipose tissue of Panc.02-tumor-bearing mice on each of the diets (*n =* 8/group). Total RNA was extracted and genomic DNA contamination was removed by using RNeasy Mini Kit (Qiagen; Valencia, CA). Total RNA was quantified by using a Nanodrop 2000 spectrophotometer (ThermoFisher Scientific), and reverse-transcribed to cDNA using the High Capacity cDNA Reverse Transcription kit (Life Technologies). Real-time qPCR was performed by using TaqMan real time PCR reagents and an Applied Biosystems StepOnePlus Real-Time PCR System (Life Technologies). Primer sequences were based on previously published studies. Real-time qPCR data were calculated using the standard curve method and normalized to 18s RNA. Relative quantification or fold change in gene expression was determined using the 2^ΔΔCt^ method using data from lean mice as reference control [41].

### 2.12. Systemic Plasma Cytokine and Metabolic Marker Analysis 

Fasting blood was collected at sacrifice, centrifuged, and plasma was stored at −80 °C. IL-6 and leptin from tumor-free (TF, *n =* 5–7/group) and Panc.02 tumor-bearing mice (*n =* 5–8/group) on each of the diets were measured using a Milliplex MAP Multiplex Assay (EMD Millipore; Billerica, MA, USA) and quantified on a Bio-plex 200 system (Bio-Rad; Hercules, CA) using Luminex-200 software (Luminex; Austin, TX, USA), per the manufacturer’s instructions. Each assay was performed in duplicate.

### 2.13. Statistical Analysis

All data were assessed for normality and equal variances, and either parametric or nonparametric analyses were used to detect differences between treatment groups. If data were skewed, transformation (log or square root) was done prior to statistical analysis. Differences in tumor weight, plasma mediators, and the distribution of cells in the spleen, TDLN, and tumor-infiltrating cells were assessed between groups via a one-way ANOVA or Kruskal–Wallis test, depending on normality and variance. A post-hoc test for trend was done to assess the linear relationship between increasing adiposity (i.e., lean, overweight, obese phenotype) and immune outcomes. Body weight, food intake, and primary tumor volume were examined using a two-way ANOVA, followed by Tukey’s or Bonferroni correction for multiple comparisons where appropriate. For survival studies, mice were counted as death for time to event analysis (survival analysis). All data are presented as the mean plus or minus the standard error of the mean. All analyses were conducted using GraphPad Prism 5 software (GraphPad Software; La Jolla, CA, USA) and statistical significance was accepted at the *p* < 0.05 level.

## 3. Results

### 3.1. The Panc.02 Model Is Characterized by a Shift Toward Suppressive Myeloid-Lineage Cells 

**Figure 1:**
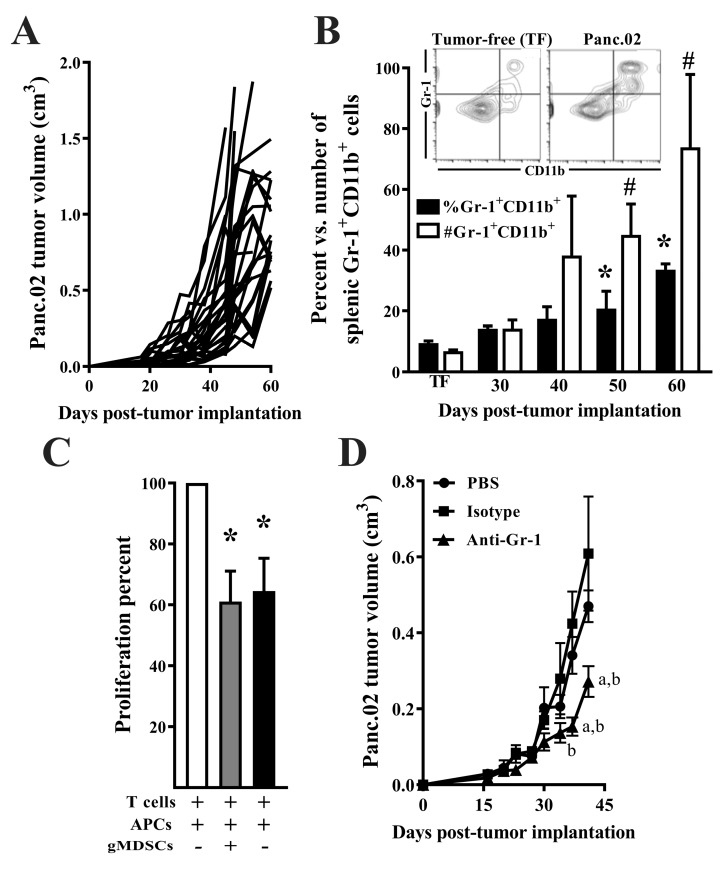
Tumor growth and the accumulation and function of suppressive myeloid cells in Panc.02 tumor-bearing mice. (**A**) Panc.02 tumor growth in C57BL/6 mice (*n =* 25). (**B**) The percent and number of splenic Gr-1^+^CD11b^+^ cells in tumor-free (TF; *n =* 8) and Panc.02 tumor-bearing mice at day 30 (*n =* 2), 40 (*n =* 7), 50 (*n =* 9), and 60 (*n =* 5) days post-tumor implantation was quantified by flow cytometry, as seen in the Figure 1B insert. Both the percent (black bars; KW = 14.90, *p* = 0.005) and number (open bars; KW = 15.73, *p* = 0.003) of splenic Gr-1^+^CD11b^+^ cells increased with tumor burden (asterisk or pound sign indicate significant difference from TF mice). (**C**) Irradiated (2000 Rads) antigen-presenting cells (APCs) and myeloid-derived suppressor cell (MDSC) subsets isolated from Panc.02 tumor-bearing mice (day 60 post-tumor implantation, *n =* 7) were mixed with isolated splenic T cells from tumor free BALB/c mice in a mixed lymphocyte reaction. Cells were incubated for five days and proliferation was measured via thymidine incorporation. Percent of maximal proliferation is reported. T cell proliferation was inhibited by gMDSCs by 38.9 ± 10.0% (*p* = 0.020) and mMDSCs by 33.8 ± 11.7% (*p* = 0.047). Asterisk indicates a significant difference from wells containing T cells + APCs without either MDSC subset. (**D**) C57BL/6 mice (*n =* 10–11/group) were implanted s.c. with 1 × 10^6^ Panc.02 cells and administered PBS, isotype control, or anti-Gr-1 antibody every three days beginning at day 16 post-tumor implantation. Depletion of Gr-1^+^ cells reduced tumor volume compared to PBS and isotype control mice (two-way ANOVA, time × group, F_(16,224)_ = 3.52, *p* < 0.001. Letters designate a significant difference from PBS (a) or isotype control antibody (b), Tukey’s multiple comparison test, *p* < 0.05).

Panc.02 tumor-bearing mice (*n =* 25) had palpable tumors by day 19 post-implantation, as seen in Figure 1A. By day 60 post-tumor implantation, the mean tumor volume in Panc.02 tumor-bearing mice was 0.90 ± 0.28 cm^3^. Splenocytes were isolated from tumor-free (TF; *n =* 8/group) and Panc.02 tumor-bearing animals at 30 (*n =* 2/group), 40 (*n =* 7/group), 50 (*n =* 9/group), and 60 (*n =* 5/group) days post-tumor implantation to characterize the time course and magnitude of the tumor-associated increase in the Gr-1^+^CD11b^+^ MDSC population. Both the percent(black bars; *p* = 0.005), and number (open bars; *p* = 0.003), of splenic Gr-1^+^CD11b^+^ MDSCs were significantly increased at day 50 and 60 post-tumor implantation, as seen in Figure 1B. 

To test the ability of MDSC subsets to inhibit T cell proliferation, T cells from BALB/c mice were cultured with APCs from Panc.02 tumor-bearing mice (*n =* 7) in a mixed lymphocyte reaction (MLR) with and without MDSC subsets from Panc.02 tumor bearing mice. Both MDSC subsets significantly suppressed the proliferative capacity of T cells, as seen in Figure 1C (*p* = 0.014). Gr-1^Hi^CD11b^+^ gMDSCs inhibited T cell proliferation by 38.9 ± 10.0% (*p* = 0.020) and Gr-1^Lo^CD11b^+^ mMDSCs inhibited T cell proliferation by 33.8 ± 11.7% (*p* = 0.047). To determine the contribution of MDSC accumulation to Panc.02 tumor progression, we examined the effect of depletion of this myeloid population on tumor growth. Depletion of Gr-1^+^ cells beginning at day 16 post-tumor implantation significantly reduced Panc.02 tumor growth compared to PBS and isotype control antibody-treated mice, as seen in Figure 1D (*p* < 0.001). 

### 3.2. Increased Adiposity Significantly Enhances Tumor Growth While Decreasing Survival in Panc.02 Tumor-Bearing Mice

Anthropometric measurements in mice following consumption of a 30% calorie-restricted diet, a 10% kcal from fat diet ad libitum, and 60% kcal from fat diet ad libitum for 16 weeks are displayed in Table 1. Data are pooled from two independent experiments, *n =* 12–14/group. Body weight, lean mass, fat mass, and percent fat were significantly different among the dietary interventions groups (one-way ANOVA *p* < 0.001 for all outcomes). Mice fed the 10% kcal from fat diet or the 60% kcal from fat diet had significantly higher body weights (*p* < 0.001), lean mass (*p* < 0.001), fat mass (*p* < 0.001), and percent body fat (*p* < 0.010) compared to the 30% CR diet. Furthermore, mice fed the 10% kcal from fat diet and the 60% kcal from fat diet differed in body weight (*p* < 0.001), fat mass (*p* < 0.001), and percent body fat (*p* < 0.001). Based on the body composition analyses, mice consuming the 30% CR diet, the 10% kcal from fat diet ad libitum, and the 60% kcal from fat diet ad libitum had significantly different percent body fat; thus, were categorized phenotypically as lean, overweight, and obese, respectively. Increasing adiposity resulted in elevated insulin, leptin, and IL-6 in both tumor free and Panc.02 tumor-bearing animals, as seen in Supplemental Figure S1.

**Figure 2:**
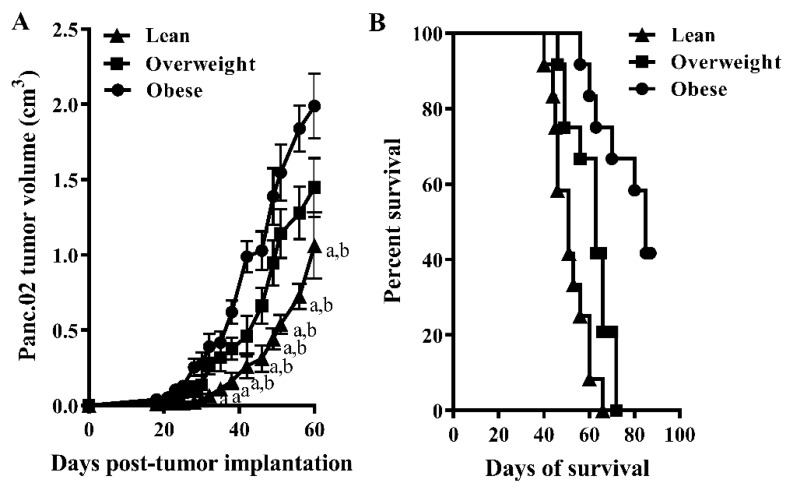
Increasing adiposity significantly enhances tumor growth while decreasing survival in Panc.02 tumor-bearing mice. (**A**) Tumor volume was significantly greater in overweight and obese compared to lean tumor-bearing mice (two-way repeated measures ANOVA, F_(50,525)_ = 1.392, time × treatment group *p* = 0.044). Letters designate a significant difference from obese (a) or overweight (b) mice, Bonferroni multiple comparison test, *p* < 0.05). (**B**) Increasing adiposity significantly reduced survival, as seen in Figure 2B (*n =* 12/group, Logrank test for trend, *p* < 0.010).

Overweight and obese mice had significantly increased tumor growth over time compared to lean mice, as seen in Figure 2A (*n =* 8/group, *p* = 0.044). Increasing adiposity also significantly reduced survival in Panc.02 tumor-bearing mice, as seen in Figure 2B (*n =* 12/group, Logrank test for trend, *p* < 0.010). Median survival was 85, 63, and 51 days for lean, overweight, and obese mice, respectively.

### 3.3. Increased Adiposity Induces the Expansion of Immunosuppressive Cell Populations and Reduces the Splenic CD8^+^ T Cell to MDSC Ratio 

**Figure 3:**
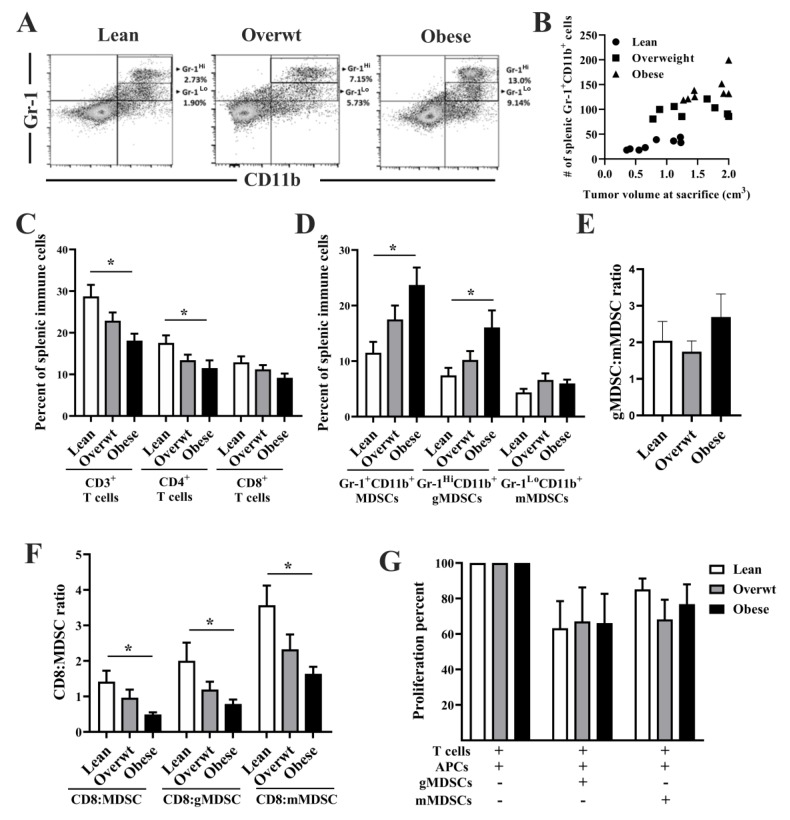
A significant linear relationship exists between increasing adiposity and the accumulation of splenic Gr-1^+^CD11b^+^ cells and a reduction in the CD8 T cell to MDSC ratio in Panc.02 tumor-bearing animals. (**A**) Representative bivariate plot using Gr-1^Hi^ Gr-1^Lo^ vs. CD11b expression in the spleen of a lean, overweight, and obese Panc.02 tumor-bearing mouse 60 days post-tumor implantation. Arrows indicate different subpopulations of MDSCs subsets (Gr-1^Hi^CD11b^+^ and Gr-1^Lo^CD11b^+^) in lean (2.71%, 1.90%), overweight (7.15% and 5.73%), and obese (13.0% and 9.14%) Panc.02 tumor-bearing animals (*n =* 7–8/group). (**B**) End tumor volume was significantly correlated with the number of splenic Gr-1^+^CD11b^+^ cells at sacrifice (Pearson’s r = 0.764, *p* < 0.001). (**C**) Increasing adiposity significantly reduced percentage of splenic CD3^+^ T cells (one-way ANOVA, F_(2,34)_ = 5.14, *p* = 0.011) and CD4^+^ T cells (one-way ANOVA; F_(2,33)_ = 3.34, *p* = 0.048) and increased the percentage of (**D**) total splenic Gr-1^+^CD11b^+^ MDSCs (one-way ANOVA, F_(2,34)_ = 5.47, *p* = 0.009) and Gr-1^Hi^CD11b^+^ gMDSCs (one-way ANOVA, F_(2,34)_ = 4.52, *p* = 0.017). (**E**) No significant difference was observed in splenic gMDSC:mMDSC ratio. Increasing adiposity resulted in a significantly lower (**F**) CD8 T cell:MDSC ratio (one-way ANOVA, F_(2,33)_ = 3.37, *p* = 0.046), CD8 T cell:gMDSC ratio (one-way ANOVA; F_(2,33)_ = 3.34, *p* = 0.049), and CD8 T cell:mMDSC ratio (one-way ANOVA, F_(2,33)_ = 4.67, *p* = 0.016). Asterisks indicate a significant post-hoc test for trend based on body composition. (**G**) Both granulocytic and monocytic MDSCs reduced T cell proliferation (one-way ANOVA, F_(1,22)_ = 6.88; *p* = 0.010); however, no significant differences were observed in the suppressive capacity of MDSC subsets on a per cell basis among lean, overweight, and obese mice.

A bivariate plot of splenic MDSC subsets using Gr-1^Hi^/Gr-1^Lo^ vs. CD11b expression, as seen in Figure 3A, is shown from a representative lean, overweight, and obese Panc.02 tumor-bearing mouse. The total number of splenic Gr-1^+^CD11b^+^ MDSCs was correlated with tumor volume at sacrifice, as seen in Figure 3B (Pearson’s r = 0.764, *p* < 0.001). The percentage of total splenic CD3^+^ T cells (*p* = 0.011) and splenic CD4^+^ T cells, as seen in Figure 3C.

Figure 3C (*p* = 0.048) was significantly reduced with increasing adiposity in a linear relationship (post-hoc test for trend, *p* = 0.003 and *p* = 0.017, respectively). The percent of splenic CD8^+^ T cells was reduced with increasing adiposity, although this did not reach statistical significance. Increased adiposity significantly increased the percentage of total splenic Gr-1^+^CD11b^+^ MDSCs (*p* = 0.009) and Gr-1^Hi^CD11b^+^ gMDSCs (*p* = 0.017), as seen in Figure 3D. No differences emerged between groups in the percentage of Gr-1^Lo^CD11b^+^ mMDSCs, as seen in Figure 3D, or in the splenic gMDSC:mMDSC ratio, as seen in Figure 3E. However, a significantly reduced CD8^+^ T cell:MDSC ratio (*p* = 0.046), CD8 T cell:gMDSC ratio (*p* = 0.049), and CD8:mMDSC ratio (*p* = 0.016) was observed with increasing adiposity, as seen in Figure 3F. Both granulocytic and monocytic MDSCs reduced T cell proliferation (one-way ANOVA; F_(1,22)_ = 6.88; *p* = 0.010); however, no significant differences were observed in the suppressive capacity of MDSC subsets on a per cell basis among lean, overweight and obese mice, as seen in Figure 3G. No significant differences were observed in other splenic immune cell populations among lean, overweight, and obese mice, as seen in Supplemental Table S3.

### 3.4. Increased Adiposity Induces the Expansion of Immunosuppressive Cell Populations and Reduces the CD8^+^ T Cell to MDSC Ratio in the Tumor-Draining Lymph Node and Tumor Microenvironment

**Figure 4:**
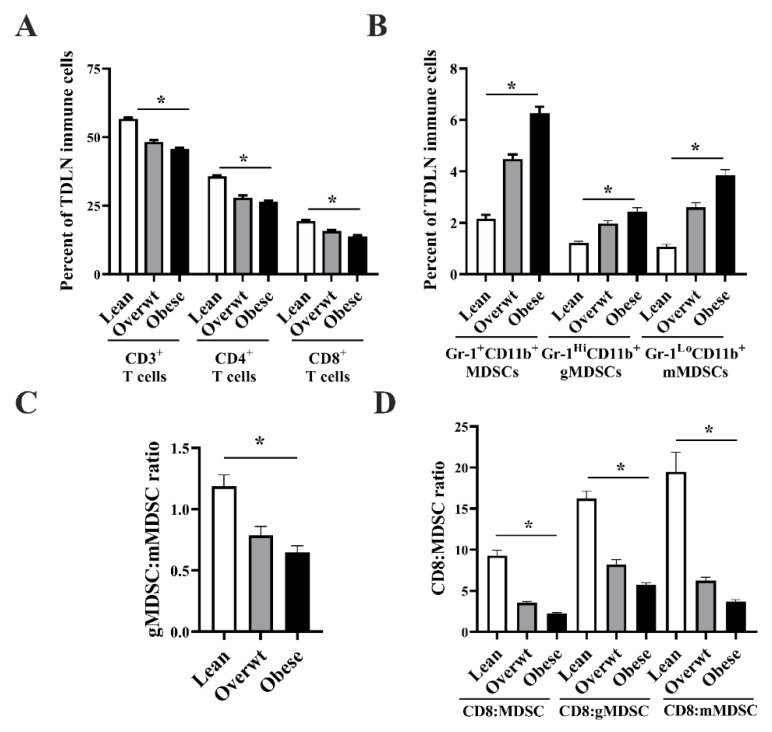
A significant linear relationship exists between increasing adiposity and the accumulation of TDLN Gr-1^+^CD11b^+^ cells and a reduction in the CD8 T cell to MDSC ratio in Panc.02 tumor-bearing animals. **(A)** Increased adiposity significantly reduced the percentage of CD3^+^ (*n =* 8/group; one-way ANOVA; F_(2,21)_ = 109.50, *p* < 0.001), CD4^+^ (one-way ANOVA; F_(2,21)_ = 73.75, *p* < 0.001), and CD8^+^ (one-way ANOVA; F_(2,21)_ = 43.72, *p* < 0.001) TDLN T cells. **(B)** Increased adiposity significantly increased Gr-1^+^CD11b^+^ MDSCs (one-way ANOVA; F_(2,21)_ = 106.10, *p* < 0.001), Gr-1^Hi^CD11b^+^ gMDSCs (one-way ANOVA; F_(2,21)_ = 31.07, *p* < 0.001), and Gr-1^Lo^CD11b^+^ mMDSCs (one-way ANOVA, F_(2,21)_ = 69.45, *p* < 0.001) in the TDLN. **(C)** Increasing adiposity shifted the TDLN gMDSC:mMDSC ratio to favor the presence of mMDSCs (one-way ANOVA, F_(2,21)_ = 13.94, *p* < 0.001). **(D)** Increasing adiposity significantly lowered the TDLN CD8 T cell:MDSC ratio (one-way ANOVA, F_(2,21)_ = 88.60, *p* < 0.001), CD8 T cell:gMDSC ratio (one-way ANOVA, F_(2,21)_ = 119.70, *p* < 0.001), and CD8 T cell:mMDSC ratio (one-way ANOVA, F_(2,21)_ = 36.35, *p* < 0.001). Asterisks indicate a significant post-hoc test for trend based on body composition (*p* < 0.001).

The percentage of TDLN CD3^+^ (*n =* 8/group; *p* < 0.001), CD4^+^ 4A (*p* < 0.001), and CD8^+^ (*p* < 0.001) T cells was significantly reduced with increasing adiposity, as seen in Figure 4A. Increased adiposity significantly increased Gr-1^+^CD11b^+^ MDSCs (*p* < 0.001), Gr-1^Hi^CD11b^+^ gMDSCs (*p* < 0.001), and Gr-1^Lo^CD11b^+^ mMDSCs (*p* < 0.001) in the TDLN, as seen in Figure 4B. Increasing adiposity shifted the TDLN gMDSC:mMDSC ratio to favor the presence of mMDSCs, as seen in Figure 4C (*p* < 0.001). A reduction in the TDLN CD8 T cell:MDSC ratio (*p* < 0.001), CD8 T cell:gMDSC ratio (*p* < 0.001), and CD8 T cell:mMDSC ratio (*p* < 0.001) was observed with increased adiposity, as seen in Figure 4D. No significant differences were observed in other TDLN immune cell populations among lean, overweight, and obese mice, as seen in Supplemental Table S4. 

**Figure 5:**
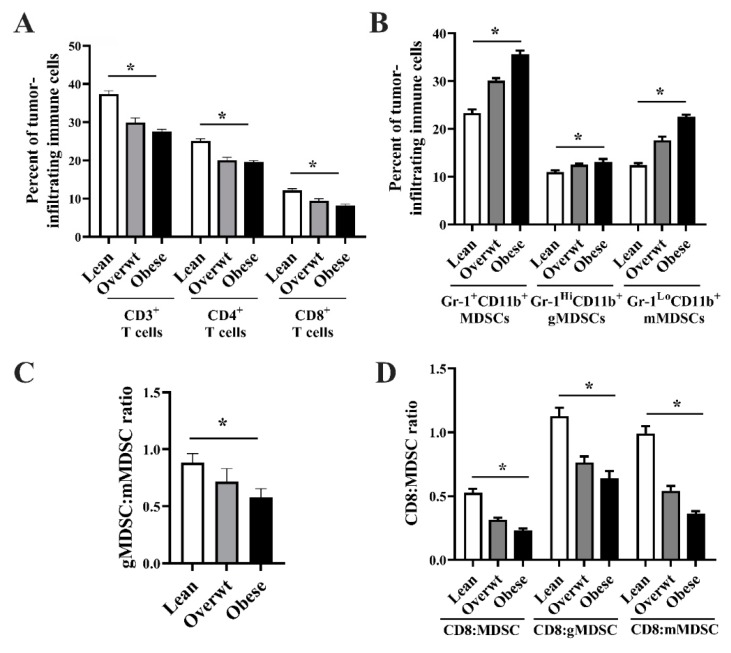
A significant linear relationship exists between increased adiposity and an increase in the accumulation of tumor infiltrating Gr-1^+^CD11b^+^ cells and a decrease in the CD8 T cell to MDSC ratio in Panc.02 tumor-bearing animals. **(A)** Increased adiposity significantly reduced the percentage of CD3^+^ (*n =* 8/group; one-way ANOVA; F_(2,21)_ = 30.91, *p* < 0.001,), CD4^+^ (one-way ANOVA; F_(2,21)_ = 27.76, *p* < 0.001), and CD8^+^ (one-way ANOVA; F_(2,21)_ = 20.05, *p* < 0.001) T cells in the tumor. **(B)** Increased adiposity significantly increased Gr-1^+^CD11b^+^ MDSCs (one-way ANOVA, F_(2,21)_ = 76.62, *p* < 0.001), Gr-1^Hi^CD11b^+^ gMDSCs (one-way ANOVA, F_(2,21)_ = 5.88, *p* = 0.009), and Gr-1^Lo^CD11b^+^ mMDSCs (one-way ANOVA, F_(2,21)_ = 88.25, *p* < 0.001) in the tumor. **(C)** Increasing adiposity shifted the gMDSC:mMDSC ratio in the tumor to favor the presence of mMDSCs (on one-way ANOVA, F_(2,21)_ = 23.16, *p* < 0.001). **(D)** Increasing adiposity significantly lowered the TDLN CD8 T cell:MDSC ratio (one-way ANOVA, F_(2,21)_ = 46.74, *p* < 0.001), CD8 T cell:gMDSC ratio (one-way ANOVA, F_(2,21)_ = 19.40, *p* < 0.001), and CD8 T cell:mMDSC ratio (one-way ANOVA, F_(2,21)_ = 64.24). Asterisks indicate a significant post-hoc test for trend based on body composition (*p* < 0.010).

The percentage of tumor-infiltrating CD3^+^(*n =* 8/group; *p* < 0.001), CD4^+^ (*p* < 0.001), and CD8^+^ (*p* < 0.001) T cells was significantly reduced with increasing adiposity, as seen in Figure 5A. Increasing adiposity resulted in a significant increase in tumor-infiltrating Gr-1^+^CD11b^+^ MDSCs (*p* < 0.001), Gr-1^Hi^CD11b^+^ gMDSCs 5B (*p* = 0.009) and Gr-1^Lo^CD11b^+^ mMDSCs (*p* < 0.001), as seen in Figure 5B. Increased adiposity shifted the tumor-infiltrating gMDSC:mMDSC ratio to favor the presence of mMDSCs, as seen in Figure 4C (*p* < 0.001). A reduction in the tumor-infiltrating CD8 T cell:MDSC ratio (*p* < 0.001), CD8 T cell:gMDSC ratio (*p* < 0.001), and CD8 T cell:mMDSC ratio, as seen in Figure 4D (*p* < 0.001) was observed with increased adiposity.

### 3.5. Increased Adiposity Induces the Expansion of Immunosuppressive Cell Populations in Adipose Tissue of Panc.02 Tumor-Bearing Animals and Immune Compartments in Tumor-Free Mice

**Figure 6:**
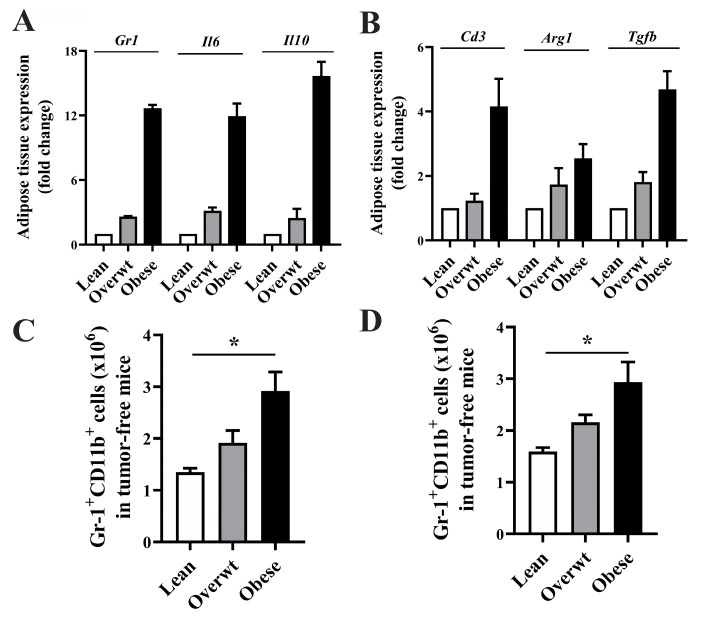
Increasing adiposity induces the upregulation of inflammatory and MDSC-associated markers in the adipose tissue of tumor-bearing mice and induces the expansion of immunosuppressive cell populations in immune compartments in tumor-free mice. The abundance of immune-related (**A**,**B**) genes within the adipose tissue microenvironment was increased in obese Panc.02 tumor-bearing animals (*n =* 8/group). In tumor-free mice, increased adiposity significantly increased the percentage of (**C**) inguinal lymph node (one-way ANOVA, F_(2,15)_ = 9.48, *p* = 0.002) and (**D**) splenic (one-way ANOVA, F_(2,15)_ = 8.190, *p* = 0.001) Gr-1^+^CD11b^+^ MDSCs. Asterisks indicate a significant post-hoc test for trend based on body composition.

The abundance of *Gr1*, *Il6*, and *Il10*, which encode Gr-1, the myeloid differentiation marker found on granulocytes, macrophages, and MDSCs, and the cytokines IL-6 and IL-10, which can contribute to the recruitment of MDSCs [42], was elevated with increasing adiposity in the adipose tissue of Panc.02 tumor-bearing mice, as seen in Figure 6A. Furthermore, the abundance of Cd3, Arg1, and Tgfb, which encode the T cell co-receptor CD3, arginase-1, an enzyme found in immunosuppressive myeloid cells [43], and the immunoregulatory cytokine, transforming growth factor-beta (TGF-β) that can be released from MDSCs to inhibit T cell function [42], was elevated with increasing adiposity in the adipose tissue of Panc.02 tumor-bearing mice, as seen in Figure 6B. In tumor-free mice, increased adiposity significantly increased the percentage of inguinal lymph node MDSCs, as seen in Figure 6C (*p* = 0.002) and splenic (*p* = 0.001) Gr-1^+^CD11b^+^ MDSCs, as seen in Figure 6D.

## 4. Discussion

We demonstrated that Panc.02 tumor-bearing mice accumulate immunosuppressive MDSCs in multiple sites, including spleen, tumor draining lymph node, tumor, and adipose tissue. Antibody depletion of Gr-1^+^ MDSCs significantly reduced Panc.02 tumor growth demonstrating a functional role for MDSCs in tumor progression. We demonstrated that as we increased adiposity across the continuum of lean, overweight, and obesity, we increased pancreatic tumor growth and decreased survival in a dose-dependent manner. We also observed a significant linear relationship between increasing adiposity and both the accumulation of Gr-1^+^CD11b^+^ cells, and a reduction in the CD8 T cell to MDSC ratio in multiple lymphoid organs and within the TME in Panc.02 tumor-bearing animals. Furthermore, the increase in adiposity resulted in elevated inflammatory and immunosuppressive myeloid markers in the adipose tissue in Panc.02 tumor-bearing mice and increased the expansion of Gr-1^+^CD11b^+^ cells in secondary lymphoid organs in the absence of tumor. These data suggest that increasing adiposity may be creating an immunosuppressive environment in key tissues during and prior to tumor development.

Both clinical and preclinical models investigating the balance between tumor-promoting/immunosuppressive factors and protective antitumor mechanisms critical for influencing cancer progression often fail to assess the contributions of common modifying factors, such as age and increased adiposity. In 2016, 39% of adults were overweight and 13% were obese worldwide [44,45] and obesity is a major risk factor for pancreatic cancer [2]; thus, it is imperative to understand both the direct and indirect effects of obesity on cancer progression [46]. Obesity is a pathological state associated with dysregulated inflammatory and metabolic mediators, including blood glucose, insulin, IGF-1, and leptin [47]. Leptin, an adipokine correlated with adipose tissue mass [48], is elevated in people with obesity and obese mice, an observation confirmed in the current study, and may play a cancer promoting role in obesity-related cancers [15]. Clinically, high prediagnostic levels of serum leptin are associated with an increase in the risk of pancreatic cancer (odds ratio = 2.55 (95% CI, 1.23–5.27) [49]. Both diet-induced obesity and models of genetic obesity show increases in serum leptin, concurrently with increased tumor growth and metastasis in murine models of pancreatic cancer [50,51,52]. Conversely, the progression from intraepithelial lesions to PDAC is delayed in mice administered a prolonged calorie restriction diet in both orthotopic and genetic models of pancreatic cancer [35]. Previous reports, and the current study, suggest that lean mice have slower tumor growth and better survival outcomes than obese mice; furthermore, in the current study, we demonstrate that there is a linear relationship between adiposity and these outcomes. These data in murine models of pancreatic cancer are consistent with the epidemiological data demonstrating a link between obesity and pancreatic risk and mortality [53,54]. 

Our data demonstrate that increasing adiposity decreased the percentage of total CD3^+^ T cells, CD4^+^ helper T cells, and CD8^+^ cytotoxic T cells in the spleen, tumor draining lymph node, and TME. Similar to previous reports detailing T cell impairments in response to obesity [55], these data demonstrate that obesity can alter the percentage of lymphoid cells across multiple tissue types. However, we also demonstrate that the observed effects on immune cell distribution are linearly related to the degree of adiposity, with overweight mice having an intermediate phenotype between lean and obese mice. In addition to obesity-induced impairments in antitumor immunity, increasing adiposity stimulates tumor-derived immunosuppressive factors to drive the expansion of suppressive myeloid lineage cells, including MDSCs. MDSCs are early myeloid-lineage cells and precursors of macrophages, dendritic cells, and granulocytes [56]. Murine MDSCs are comprised of two subsets, granulocytic (Gr-1^Hi^CD11b^+^) MDSCs and a monocytic (Gr-1^Lo^CD11b^+^) MDSCs, each contributing to disease progression by suppressing both innate and adaptive antitumor immune responses through multiple mechanisms [23,57]. Similar to previous preclinical subcutaneous pancreatic [58] and orthotopic renal [59] models, we demonstrate that with increasing adiposity, an expansion of total MDSCs, gMDSCs, and mMDSCs occurred in the spleen, tumor draining lymph node, and TME. We also noted a preferential expansion of mMDSCs within the TME in overweight and obese mice, suggesting that increased adiposity can alter MDSC subset distribution in a tissue specific manner. However, similar to previous reports [59], splenic MDSC subsets from all dietary groups were equally suppressive on a per cell basis. While equally suppressive, prevention of the accumulation of MDSCs by the maintenance of a lean phenotype may be physiologically important and suggestive of prolonged antitumor immunity. 

Clinically, MDSCs accumulate in pancreatic cancer patients, and their accumulation correlates with disease progression [60,61]. The accumulation of MDSCs in cancer patients is also associated with a concurrent reduction in CD8^+^ T cells [62]. However, the impact of increased adiposity on the CD8 T cell to MDSC ratio in cancer patients remains unknown, but may be related to clinical outcomes in numerous cancer types. In the current study, increased adiposity induced an unfavorable shift in the CD8^+^ T cell to MDSC ratio in multiple lymphoid organs and within the TME. This reduction in effector T cells that occurs concurrently with an increase in immunosuppressive MDSCs may provide a robust inhibitory signal to prevent immune recognition of tumor antigens and immune-mediated tumor clearance.Thus, overweight and obesity-induced accumulation of MDSCs may contribute significantly to the exacerbation of tumor development. These data suggest that the maintenance of body weight over time, as observed in lean mice, has a protective effect on immune and tumor outcomes. 

Obesity-induced alterations in myeloid markers were not limited to classic lymphoid organs or the TME. We observed increased gene expression of *Gr1*, *Il6*, and *Il10* in the adipose of obese mice, suggesting an obesity-induced expansion in myeloid markers and cytokines that may promote the accumulation of immunosuppressive MDSCs [43]. An increase in the gene expression of *Cd3*, *Arg1*, and *Tgfb* was observed in adipose tissue of obese mice further demonstrating an abundance of immune cell markers and immune suppressive signaling pathways associated with myeloid cells [42]. The role of Gr-1^+^CD11b^+^ cells within adipose tissue may have opposing effects in tumor-free vs. tumor-bearing hosts. Xia, et al. [63] report that Gr-1^+^CD11b^+^ cells accumulate in the adipose of genetically obese (ob/ob) tumor-free animals in comparison to wild-type controls, and improve insulin sensitivity and reduce serum IL-6, suggesting that MDSCs may be negative regulators of obesity-induced inflammation in the absence of tumor. Our data demonstrate that in pancreatic tumor-bearing animals, increased adiposity results in the accumulation of myeloid-lineage and immunosuppressive factors in adipose tissue. Further studies are needed to determine the role of myeloid-lineage cells within the adipose tissue of tumor-bearing hosts. 

Obesity is associated with alterations in the infiltration and accumulation of immune cells into adipose tissue that further contribute to chronic inflammation and dysregulated metabolism [64]. To separate the effects of obesity-induced inflammation from tumor-derived factors, which can stimulate MDSC expansion and accumulation, we examined the effect of increased adiposity on myeloid-lineage cells in tumor-free animals. Similar to a previous report demonstrating MDSC accumulation in the bone marrow, blood, spleen, adipose tissue, and liver of genetically obese (ob/ob), tumor-free animals [63], we observed increased Gr-1^+^CD11b^+^ cells in immune organs from diet-induced overweight and obese mice in the absence of tumor. These data suggest that increased adiposity may be creating a proinflammatory and immunosuppressive environment prior to tumor development. Ma et al., demonstrate that MDSCs collected from patients with premalignant lesions were similar in subpopulation composition and immunosuppressive capacity to MDSCs collected from cancer patients, and that levels of MDSC in premalignancy correlate negatively with in vivo effector responses [65]. Thus, it may be that increasing adiposity in the absence of tumor contributes significantly to immune suppression via the accumulation of immunosuppressive MDSCs, which create an environment conducive to tumor initiation and/or growth. However, the full extent to which MDSCs and the inflammatory environment associated with increased adiposity contribute to cancer progression and/or response to therapy remains unclear.

## 5. Conclusions

In conclusion, we observed a linear relationship between increased adiposity, enhanced tumor growth, and decreased survival. These changes occurred concurrently with an expansion of immunosuppressive cells in lymphoid organs and within the TME, as well as increased expression of suppressive and inflammatory markers within the adipose tissue of tumor-bearing mice. Furthermore, increased adiposity expanded myeloid-lineage cells in the absence of tumor, demonstrating that obesity-induced alterations in myeloid populations can occur within secondary lymphoid organs. These results contribute to our understanding of the role of increasing adiposity on inflammatory and tumor-associated immunosuppressive factors. Understanding the detrimental effects of increased adiposity on the emergence of immunosuppressive cell types and identifying agents that block this expansion may reduce cancer progression and improve response rates to current and emerging therapies in patients diagnosed with numerous cancer types.

## Figures and Tables

**Table 1:** Body composition measures (mean ± SEM) of mice on the three dietary treatment regimens (*n =* 12–14/group).

Treatment Group	Body Weight (g)	Lean Mass (g)	Fat Mass (g)	% Fat
30% CR (lean)	18.9 ± 0.2	13.1 ± 0.3	3.9 ± 0.2	23.2 ± 0.7
10% kcal from fat (overweight)	25.9 ± 0.7 ^a^	16.7 ± 0.3 ^a^	7.9 ± 0.6 ^a^	31.5 ± 1.5 ^a^
60% kcal from fat (obese)	35.1 ± 1.5 ^a,b^	16.4 ± 0.5 ^a^	18.2 ± 1.0 ^a,b^	49.0 ± 2.3 ^a,b^

^a^ significantly different from 30% calorie-restricted (CR) fed mice (Bonferroni correction *p* < 0.010); ^b^ significantly different from 10% kcal fat fed mice (Bonferroni correction *p* < 0.001).

## Supplementary Materials

The following are available online at https://www.mdpi.com/2072-6643/11/12/3012/s1, Table S1: Diet Formulations; Table S2: List of antibodies for flow cytometry; Table S3: Effect of increased adiposity on splenic immune cell Distribution; Table S4: Effect of increased adiposity on TDLN immune cell distribution; Figure S1. Metabolic and inflammatory mediators in the plasma of tumor free and Panc.02 tumor-bearing mice. Plasma levels of (A, D) insulin (B, E) leptin and (C, F) IL-6 were measured in TF (*n =* 5–7/group) and Panc.02 tumor-bearing mice day at 60 day post-tumor implantation (*n =* 5–8/group) using the Milliplex MAP Multiplex Assay, respectively. Adiposity increased plasma insulin (F_(2,12)_ = 6.71, *p* = 0.011, log rank test for trend *p* = 0.003), leptin (F_(2,12)_ = 19.50, *p* < 0.001, log rank test for trend *p* < 0.001), and IL-6 (F_(2,12)_ = 15.56, *p* < 0.001, log rank test for trend *p* < 0.001) in tumor free mice (A–C). Adiposity increased plasma insulin (F_(2,17)_ = 3.95, *p* = 0.039, log rank test for trend *p* = 0.014), leptin (F_(2,17)_ = 15.16, *p* < 0.001, log rank test for trend *p* < 0.001), and IL-6 (F_(2,17)_ = 3.59, *p* = 0.048, log rank test for trend *p* = 0.019) in tumor-bearing mice (D–F).

## Abbreviations

APC: antigen presenting cell; BMI, body mass index; DC, dendritic cell; IL, Interleukin; i.p., intraperitoneal, MDSC, myeloid-derived suppressor cell; MLR, mixed lymphocyte reaction; NK, natural killer; s.c., subcutaneous; TDLN, tumor-draining lymph nodes; TF, tumor-free.
