# Supplementary material for: Increased Adiposity Enhances the Accumulation of MDSCs in the Tumor Microenvironment and Adipose Tissue of Pancreatic Tumor-Bearing Mice and in Immune Organs of Tumor-Free Hosts
Source: Nutrients. 2019 Dec 10;11(12):3012. doi: 10.3390/nu11123012 (PMC6950402; doi:10.3390/nu11123012)
Supplement: Supplementary file 1 [file nutrients-11-03012-s001.pdf]

Supplemental Table 1. Diet Formulations

Class description	Ingredient (grams)	30% calorie restricted diet	10% Kcal from fat diet	60% Kcal from fat diet
Protein	Casein, 80 Mesh	200	200	200
Protein	L-Cystine	3	3	3
Carbohydrate	Corn Starch	197.9	315	0
Carbohydrate	Maltodextrin 10	0 g	35	125
Carbohydrate	Sucrose	197.9	350	68.8
Fiber	Cellulose	50	50	50
Fat	Soybean Oil	25	25	25
Fat	Lard	20	20	245
Mineral	RD96 Mineral Mix	10	10	10
Mineral	Dicalcium Phosphate	13	13	13
Mineral	Calcium Carbonate	5.5	5.5	5.5
Mineral	Potassium Citrate	16.5	16.5	16.5
Vitamin	Choline Bitartrate	2	2	2
Vitamin	AIN-76A vitamin mix	10	10	10
Dye	Yellow FD&C #5	0	0.05	0
Dye	Blue FD&C #1	0	0	0.05
	Total:	750.8	1055.05 g	773.85
**Caloric Information Physiological Fuel Values**				
Protein (kcal)		29%	20%	20%
Fat (kcal)		14%	10%	60%
Carbohydrate (kcal)		57%	70%	20%
Energy Density (kcal/gm)		3.78	3.85	5.24

Supplemental Table 2. List of antibodies for flow cytometry.

Antibody	Clone	Fluorochrome	Catalog number	Supplier
CD3ε	145-2C11	FITC, APC-Cy7	553062, 557596	BD Biosciences
CD4	GK1.5	PE	553730	BD Biosciences
CD8α	53-6.7	APC	553035	BD Biosciences
CD11b	M1/70	APC-Cy7	557657	BD Biosciences
CD11c	HL3	PE	553802	BD Biosciences
CD19	1D3	FITC	553785	BD Biosciences
CD161 (NK1.1)	PK136	PE	557391	BD Biosciences
F4/80	BM8	APC	17-4801-82	eBiosciences
Fc block	93	X	101302	Biolegend
Gr-1	RB6-8C5	FITC	553127	BD Biosciences
I-Ab	AF6-120.1	FITC	553551	BD Biosciences
Ly6C	1A8	APC	560599	BD Biosciences
Ly6G	AL-21	APC-Cy7	560596	BD Biosciences

Supplemental Table 3. Effect of increased adiposity on splenic immune cell distribution

A Splenic immune cells (percent)	Lean (n=12)	Overwt (n=14)	Obese (n=10)	p-value
B cells ($CD19^+$)	45.7 ± 8.8	41.6 ± 9.2	43.4 ± 7.4	0.487
Total T cells ($CD3^+$)	28.7 ± 10.0	22.9 ± 7.4	18.1 ± 5.2[a]	0.011
Helper T cells ($CD3^+CD4^+$)	17.6 ± 6.3	13.4 ± 5.0	11.5 ± 5.8[a]	0.028
Cytotoxic T cells ($CD3^+CD8^+$)	12.9 ± 5.0	11.2 ± 3.8	9.2 ± 3.2	0.121
NK cells ($NK1.1^+$)	7.8 ± 3.3	6.5 ± 3.9	8.7 ± 4.8	0.529
Dendritic Cells ($I\text{-}Ab^+CD11c^+$)	10.8 ± 4.2	11.1 ± 4.9	10.6 ± 3.6	0.988
Macrophages ($I\text{-}Ab^+CD11b^+$)	12.5 ± 4.0	14.9 ±3.9	15.0 ± 2.6	0.169
MDSCs ($Gr\text{-}1^+CD11b^+$)	13.1 ± 7.7	17.5 ± 9.3	21.7 ± 11.1	0.084
gMDSCs ($Gr\text{-}1^{hi}CD11b^+$)	8.2 ± 6.3	10.2 ± 6.0	15.1 ± 10.4	0.086
mMDSCs ($Gr\text{-}1^{lo}CD11b^+$)	4.4 ± 2.3	6.6 ± 4.4	6.0 ± 2.1	0.128

B Splenic immune cells ($x10^6$)	Lean (n=12)	Overwt (n=14)	Obese (n=10)	p-value
Splenocyte count	143.5 ± 68.6	177.4 ± 109.0	189.5 ± 50.2	0.381
B cells ($CD19^+$)	69.1 ± 35.5	68.1 ± 32.7	82.4 ± 23.9	0.506
Total T cells ($CD3^+$)	37.1 ± 14.5	35.2 ± 17.4	33.9 ± 12.6	0.876
Helper T cells ($CD3^+CD4^+$)	24.0 ± 10.1	21.4 ± 12.4	22.2 ± 13.2	0.853
Cytotoxic T cells ($CD3^+CD8^+$)	17.5 ± 7.5	18.0 ± 11.0	17.8 ± 8.9	0.991
NK cells ($NK1.1^+$)	10.8 ± 7.2	9.5 ± 6.5	16.3 ± 11.3	0.136
Dendritic Cells ($I\text{-}Ab^+CD11c^+$)	15.6 ± 10.8	17.8 ± 11.0	20.7 ± 10.5	0.415
Macrophages ($I\text{-}Ab^+CD11b^+$)	17.9 ± 10.9	25.7 ± 13.8	28.5 ± 9.7	0.092
MDSCs ($Gr\text{-}1^+CD11b^+$)	21.4 ± 18.1	35.0 ± 32.5	39.4 ± 17.6	0.112
gMDSCs ($Gr\text{-}1^{hi}CD11b^+$)	13.7 ± 12.5	21.2 ± 20.6	26.4 ± 13.3	0.102
mMDSCs ($Gr\text{-}1^{lo}CD11b^+$)	6.9 ± 6.4	12.6 ± 12.0	11.7 ± 5.9	0.169

a = significantly different than Lean ($p<0.05$)

b = significantly different than Overwt ($p<0.05$)

Supplemental Table 4. Effect of increased adiposity on TDLN immune cell distribution

A TDLN immune cells (percent)	Lean (n=8)	Overwt (n=8)	Obese (n=8)	p-value
B cells ($CD19^+$)	33.8 ± 2.7	35.1 ± 2.5	35.7 ± 1.7	0.298
Total T cells ($CD3^+$)	56.7 ± 1.2	48.3 ± 2.0[a]	45.7 ± 1.3[a,b]	<0.001
Helper T cells ($CD3^+CD4^+$)	35.7 ± 0.9	28.0 ± 2.3[a]	26.4 ± 1.4[a]	<0.001
Cytotoxic T cells ($CD3^+CD8^+$)	19.4 ± 1.3	15.8 ± 1.1[a]	13.8 ± 1.3[a,b]	<0.001
Dendritic Cells ($I\text{-}Ab^+CD11c^+$)	0.6 ± 0.1	0.7 ± 0.1	0.7 ± 0.1	0.433
Macrophages ($I\text{-}Ab^+CD11b^+$)	1.1 ± 0.3	1.5 ± 0.4	2.2 ± 0.6[a,b]	<0.001
MDSCs ($Gr\text{-}1^+CD11b^+$)	2.2 ± 0.4	4.5 ± 0.5[a]	6.3 ± 0.7[a,b]	<0.001
gMDSCs ($Gr\text{-}1^{hi}CD11b^+$)	1.2 ± 0.2	2.0 ± 0.3[a]	2.4 ± 0.4[a]	<0.001
mMDSCs ($Gr\text{-}1^{lo}CD11b^+$)	1.1 ± 0.3	2.6 ± 0.5[a]	3.6 ± 0.6[a,b]	<0.001

B TDLN immune cells ($x10^6$)	Lean (n=8)	Overwt (n=8)	Obese (n=8)	p-value
B cells ($CD19^+$)	3.7 ± 0.6	4.2 ± 0.4	4.3 ± 0.3[a]	0.039
Total T cells ($CD3^+$)	6.3 ± 0.8	5.8 ± 0.8	5.6 ± 0.5	0.125
Helper T cells ($CD3^+CD4^+$)	4.0 ± 0.5	3.3 ± 0.6[a]	3.2 ± 0.4[a]	0.014
Cytotoxic T cells ($CD3^+CD8^+$)	2.2 ± 0.3	1.9 ± 0.3	1.7 ± 0.3[a]	0.014
Dendritic Cells ($I\text{-}Ab^+CD11c^+$)	0.1 ± 0.1	0.1 ± 0.1	0.1 ± 0.1	0.153
Macrophages ($I\text{-}Ab^+CD11b^+$)	0.1 ± 0.1	0.2 ± 0.1	0.3 ± 0.1[a,b]	<0.001
MDSCs ($Gr\text{-}1^+CD11b^+$)	0.2 ± 0.1	0.5 ± 0.1[a]	0.8 ± 0.1[a,b]	<0.001
gMDSCs ($Gr\text{-}1^{hi}CD11b^+$)	0.1 ± 0.1	0.2 ± 0.1[a]	0.3 ± 0.1[a,b]	<0.001
mMDSCs ($Gr\text{-}1^{lo}CD11b^+$)	0.1 ± 0.1	0.3 ± 0.1	0.5 ± 0.1[a]	<0.001

a = significantly different than Lean ($p<0.05$)

b = significantly different than Overwt ($p<0.05$)

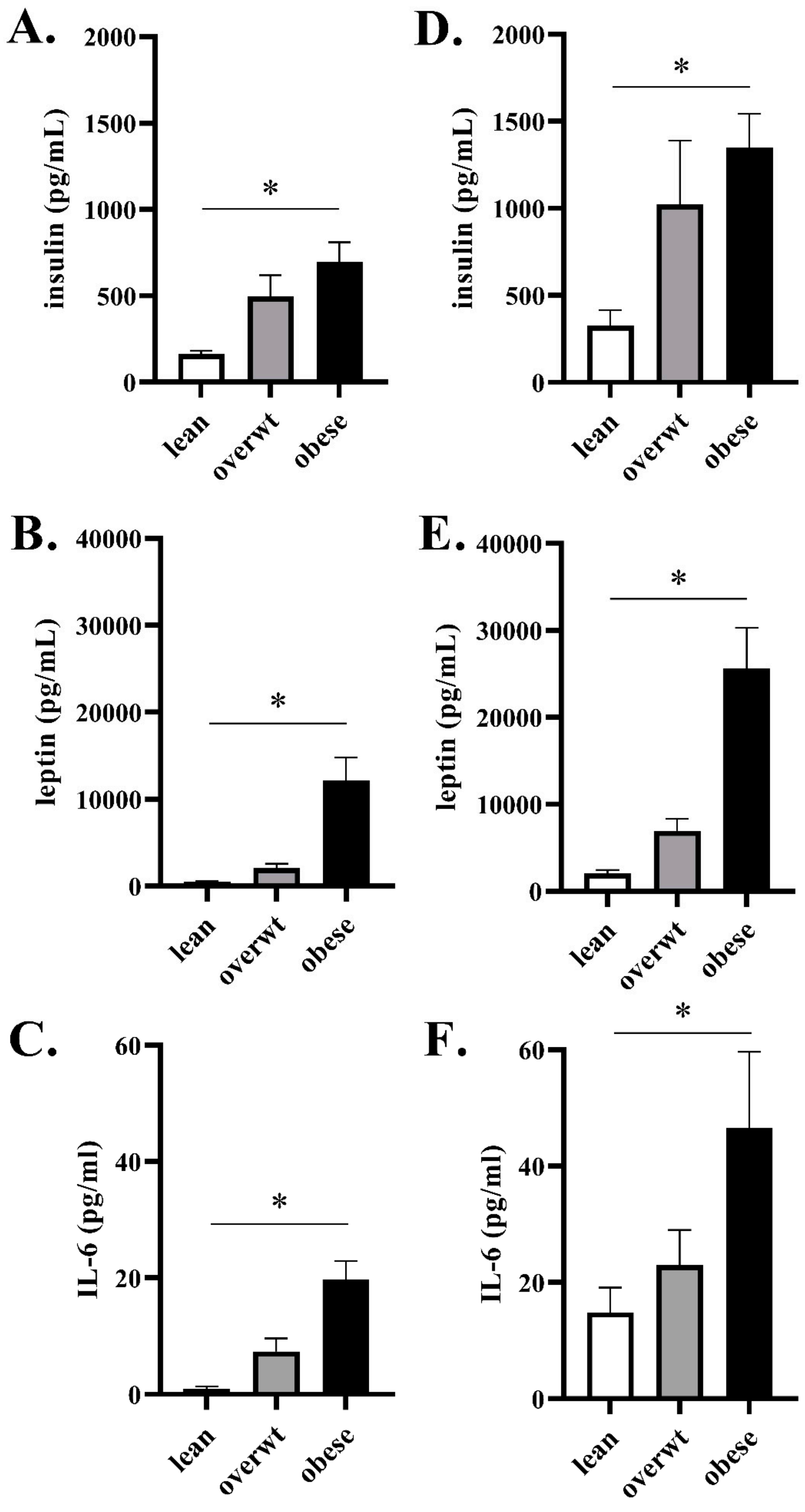
A.
insulin (pg/mL)
2000
1500
1000
500
0
*
lean
overwt
obese
D.
insulin (pg/mL)
2000
1500
1000
500
0
*
lean
overwt
obese
B.
leptin (pg/mL)
40000
30000
20000
10000
0
*
lean
overwt
obese
E.
leptin (pg/mL)
40000
30000
20000
10000
0
*
lean
overwt
obese
C.
IL-6 (pg/ml)
60
40
20
0
*
lean
overwt
obese
F.
IL-6 (pg/ml)
60
40
20
0
*
lean
overwt
obese
